# Exploiting Food-Grade Mesoporous Silica to Preserve the Antioxidant Properties of Fresh Olive Mill Wastewaters Phenolic Extracts

**DOI:** 10.3390/antiox10091361

**Published:** 2021-08-26

**Authors:** Federica Ianni, Andrea Gagliardi, Agnese Taticchi, Maurizio Servili, Nicola Pinna, Aurélie Schoubben, Roccaldo Sardella, Stefano Bruscoli

**Affiliations:** 1 Department of Pharmaceutical Sciences, University of Perugia, Via Fabretti 48, 06123 Perugia, Italy; federica.ianni@unipg.it (F.I.); nicola.pinna@studenti.unipg.it (N.P.); 2 Department of Medicine and Surgery, University of Perugia, Via Gambuli 1, 06132 Perugia, Italy; andrea.gagliardi@studenti.unipg.it (A.G.); stefano.bruscoli@unipg.it (S.B.); 3 Department of Agricultural Food and Environmental Sciences, University of Perugia, Via S. Costanzo, 06126 Perugia, Italy; agnese.taticchi@unipg.it (A.T.); maurizio.servili@unipg.it (M.S.); 4 Center for Perinatal and Reproductive Medicine, University of Perugia, Santa Maria della Misericordia University Hospital, 06132 Perugia, Italy

**Keywords:** total phenol content, total antioxidant capacity, HPLC-DAD analysis, Syloid^®^ AL-1, inclusion complex, oleacein, olive phenolic compounds, cytotoxicity, in vitro antioxidant activity, intestinal-derived epithelial cell line

## Abstract

Fresh olive mill wastewaters phenolic extracts are of great interest as preservatives or fortifying ingredients but are characterized by limited stability. The purpose of this study was to use mesoporous silica to enhance their stability and preserve their antioxidant properties. The phenolic extracts were characterized for their composition by HPLC-DAD and included in a mesoporous matrix with or without a lipid coating. The inclusion complexes were characterized in terms of total phenolic content, radical scavenging capacity and in vitro antioxidative activity and cell compatibility. Besides, inclusion complex stability under different storage conditions (22 and 37 °C, 75% relative humidity, 1 month) was evaluated. The inclusion process was nearly quantitative and modified neither the total phenolic content nor the total antioxidant capacity. None of the inclusion complex concentrations assayed on the HT29 cell line showed toxicity. Moreover, HT29 cells treated with the inclusion complex exhibited a significant antioxidant effect, while the lipid coating impaired the antioxidant activity. The complexes without lipid were stable under all the investigated conditions, while the lipid-coated products were less stable under the more drastic conditions. Overall, inclusion complexes in mesoporous silica have suitable characteristics to be used for different applications, including food supplementation.

## 1. Introduction

The demand for “specialty” or “value-added ingredients” is strongly growing, mainly for those of natural origin with functional characteristics, among which the group of phenolic compounds stands out, at present dominated by extracts from grape seeds and green tea. Nonetheless, the search for other sources of bioactive molecules is fervent and, in this regard, *Olea europaea* L. can be rightly considered as an interesting source of well-recognized biophenols, including secoiridoids such as oleuropein, demethyloleuropein, and ligstroside and their aglycons, occurring exclusively in plants from the *Oleaceae* family [1]. Indeed, according to the health claim of EU Regulation no. 432/2012, olive oil phenolic compounds are recognized as capable of contributing to the protection of blood lipids from oxidative stress if taken daily in quantities of at least 5 mg as part of moderate consumption of virgin olive oil (20 g). However, the richness of virgin olive oil (VOO) in phenolic compounds is extremely variable as a function of genetic, agronomic, and technological factors and conservation conditions of the oil, which can greatly affect its qualitative and quantitative composition in phenolic substances [2]. The daily recommended intake may thus be assured only through the consumption of oils sufficiently rich in phenolic compounds, and this does not represent a food habit observed globally due to cultural and availability factors.

Other potential opportunities for olive oil biophenols intake may lie in foods fortified with extracts from natural sources.

On the other hand, most of the phenolic substances contained in the fresh olive mill wastewaters (OMWW) are the same molecules present in virgin olive oil. In fact, in OMWW, other than small percentages of hydroxytyrosol (3,4-DHPEA) and tyrosol (p-HPEA), the dialdehydic form of decarboxymethyl elenolic acid linked to 3,4-DHPEA (3,4-DHPEA-EDA or oleacein), is usually the prevalent phenolic species, such as in VOO where, due to their high hydrophilicity, no more than 2% of the total phenolic compounds contained in the olive fruits is transferred during the mechanical separation. Additionally, verbascoside is also generally abundant in OMWW, on a cultivar-dependent basis [3]. In VOO producer countries, the volume of resulting OMWW is very large (especially in Italy, where the separation is mainly based on the high OMWW producing three-phases centrifugation system), determining the need to implement disposal practices. The growing interest in the phenolic compounds of olives and their abundance in OMWWs, up to 25 g/L, have led to the change of the traditional approach from that of the problem of waste disposal to that of waste valorization by recovering the bioactive phenolic fraction [3].

The membrane filtration method, combining ultrafiltration and reverse osmosis, has demonstrated very satisfactory selectivity and concentration capability in recovering and concentrating the OMWW phenolic fraction, giving a crude phenolic extract that can be further purified and processed for subsequent uses. Nevertheless, the multi-step treatment by membrane filtration is able to break down the polluting load, giving a permeate with a very low BOD (biochemical oxygen demand) and COD (chemical oxygen demand). This technological approach, processing fresh and previously untreated OMWW, appears very promising in comparison to other proposed methods in terms of environmental impact. Since no solvents are required for the recovery of phenolic compounds, in terms of yield, reaching the crude extract a concentration up to four times of the original OMWW, and in terms of keeping the compositional profile of the phenolic species occurring in fresh OMWW, since no spontaneous or induced hydrolyses takes place. In the last few years, a number of different uses have been proposed and tested in various food systems. In many cases, the aim was to exploit the antioxidant and/or antimicrobial activity of those phenolic extracts by pursuing higher stability, the extension of the shelf-life of the food, or the reduction or replacement of conventional preservatives. Those abilities have been successfully evaluated among others for oils, dressings, fresh or processed/cured meats, biscuits, seafood, and fish products [3,4,5,6,7], being also reputed able to contribute to the reduction of food wastes within the purposes of the circular economy model. In addition, another very promising use for phenols from OMWW consists in their addition to foods as fortifying ingredients by reason of the widely documented health-promoting properties to produce functional products [8]. Besides the great opportunity that the recovery of phenols from OMWW and their application as food ingredients represent in view of a circular economy approach, with enormous benefits for the olive oil industry in terms of added value and sustainability, there are some problems to be addressed, related to stability. In fact, in most of the investigations about the application of phenolic extracts from OMWW in food systems, when their evolution with storage has been evaluated, a variable decrease in their concentration was observed, depending on the food matrices and storage conditions [4,6,8]. In some studies, contemporary hydrolysis and oxidative degradation have been described. Furthermore, the increase of hydroxytyrosol concentration as a result of the hydrolysis of oleuropein derivatives has also been confirmed in VOO and in OMWW [5,9,10].

Stability in different food complex matrixes is a fundamental requirement for reliable bioactive ingredients, thus representing a strategic challenge in the development of new food formulations in which they must be able to guarantee the maintenance of the claimed intake and/or their functional activity.

Currently, being well documented and confirmed the functional effectiveness of the application of biophenols recovered from OMWW as fortifying or preservative ingredients, a multidisciplinary effort is needed in moving forward the development of technologies for providing “tailor-made” formulations for real exploitation opportunities.

For this purpose, to overcome the susceptibility of these compounds to adverse external effects or detrimental food processing conditions, microencapsulation represents a promising technology for improving stability and miscibility [11].

Recently, phenolic compounds extracted from grape pomace have been encapsulated in MCM-41 silica to improve their stability [12]. Along this line, here we report the preparation and complete characterization of OMWW phenolic extract inclusion complexes in mesoporous silica employed to preserve their antioxidants properties. The inclusion complexes were further processed to obtain their lipid coating and thereafter characterized as for the inclusion complexes.

According to the results obtained, the formulation process allowed the quantitative inclusion of the phenol extracts and preserved their antioxidant ability. None of the inclusion complex concentrations assayed on an intestinal-derived epithelial cell line showed toxicity. Based on the obtained results, the produced inclusion complexes in mesoporous silica have suitable characteristics to be used for different applications, including food supplementation.

## 2. Materials and Methods

### 2.1. Materials

*p*-HPEA and 3,4-DHPEA were purchased from Cabru S.A.S. (Arcore, Milan, Italy) and Fluka (Milan, Italy), respectively, while verbascoside was bought from Extrasynthese (Genay, France). 3,4-DHPEA-EDA and *p*-HPEA-EDA were extracted from VOO following a previously described method [13,14].

Lauric acid was provided by Merck Life Science (Merck KGaA, Darmstadt, Germany). Stearic acid was furnished by ACEF (Piacenza, Italy). Syloid^®^ AL-1 was a gift of Grace Davison (Worms, Germany). Syloid^®^ AL-1 is a synthetic amorphous non-ordered mesoporous silica pharmaceutical excipient (average particle size 6.5–8.1 μm, pore volume 0.4 mL/g). All other chemicals and reagents were of the highest purity grade commercially available.

The Folin–Ciocalteu reagent, 2,4,6-tris(2-pyridyl)-*s*-triazine, 6-hydroxy-2,5,7,8-tetramethyl-2-carboxylic acid, 2,2-diphenyl-1-picrylhydrazyl, hydrochloric acid, ferric chloride, sodium acetate, sodium carbonate, acetic acid, gallic acid, HPLC-grade ethanol, and HPLC-grade methanol were purchased from Merck Life Science (Merck KGaA, Darmstadt, Germany). Double distilled water (dd-H_2_O) was purified with a New Human Power I Scholar water purification system (Human Corporation, Seoul, Korea).

The cell culture reagents (RPMI medium 1640, glucose, sodium pyruvate, penicil-lin/streptomycin, and Fetal Bovin Serum (FBS)), methanol (MeOH), trypsin, Trypan Blue Solution, dichlorodihydrofluorescein diacetate (DCFDA) test were purchased from Thermo Fisher Scientific (Waltham, MA, USA). Propidium iodide (PI), sodium citrate, Triton X-100 were purchased from Merck Life Science (Merck KGaA, Darmstadt, Germany).

### 2.2. Preparation of the Phenolic Extracts and HPLC Analysis

The phenolic extracts were obtained from fresh OMWWs of olives harvested in Umbria (Central Italy) from trees of Moraiolo cultivar, as previously reported [15]. Concisely, after a hydrolytic enzymatic treatment with a pectinase/hemicellulose preparation (O-Max S, OE Italia S.r.l., Marsala, Italy) at 20 °C for 12 h, the OMWW were subjected to a procedure consisting of 3 successive membrane filtration steps (microfiltration, ultrafiltration and reverse osmosis), on a pilot-scale filtration plant. The obtained crude phenolic concentrate was subsequently treated as explained in [16] to obtain the phenolic extracts. Three different extracts were obtained: M2 and M5, obtained from the same batch of OMWW, and E from another batch of the same production season. The phenolic extracts were characterized by reversed-phase HPLC-DAD, following a previously described procedure [13]. The HPLC instrumentation was an Agilent Technologies system Mod. 1100, composed of a vacuum degasser, a quaternary pump, an autosampler, a thermostated column compartment, and DAD and equipped with a C18 column (Spherisorb ODS-1 (250 mm × 4.6 mm) 5 μm particle size, supplied by Phase Separation Ltd. (Deeside, UK)). The analytical conditions were as follows: sample volume injected 20 μL; mobile phase 0.2% acetic acid (pH 3.1) in water (solvent A)/methanol (solvent B); flow rate 1 mL/min; gradient changing as follows: 95% A/5% B for 2 min, 75% A/25% B in 8 min, 60% A/40% B in 10 min, 50% A/50% B in 16 min, and 0% A/100% B in 14 min; this last proportion was kept for 10 min and then the initial conditions were restored in 13 min; total running time 73 min. The detection of phenolic compounds was performed with a DAD at 278 nm of wavelength. Analyses were performed in duplicate.

### 2.3. Preparation and Morphological Characterization of the Inclusion Complexes

Inclusion complexes of the phenolic extracts (i.e., M2, M5, and E) and Syloid^®^ AL-1 (30:70, *w*:*w*) were prepared using the solvent evaporation method [17,18]. In particular, the phenolic extract was solubilized in 10 mL ethanol, and Syloid^®^ AL-1 was then added to the solution that was left under magnetic stirring for 2 h. Ethanol was successively evaporated under vacuum, and the powder obtained was recovered and stored in a desiccator until further use. This procedure was carried out with the different phenolic extracts to obtain the following products: Syl-M2, Syl-M5, and Syl-E.

An amount of inclusion complex was further processed to coat it with a lipid mixture (lauric acid:stearic acid, 77:33, w:w) characterized by a melting temperature of 37 °C. Briefly, the lipid mixture was melted at 40 °C, and the inclusion complex was added and stirred for 10 min [18,19]. The lipid:inclusion complex weight ratio was 1.1:1. Then, the suspension was cooled slowly by continuous stirring to give the final products: Lip-Syl-M2, Lip-Syl-M5, and Lip-Syl-E. The final products were then sieved using a 180-mesh stainless sieve. Lipid- and non-lipid-coated inclusion complexes were stored at −20 °C until further use.

Syloid^®^ AL-1 and the inclusion complex (i.e., Syl-E) morphology was investigated by scanning electron microscopy (SEM) using a field-emission microscope (Zeiss LEO 1525 equipped with a GEMINI column, Oberkochen, Germany). Samples were prepared by placing powder onto an aluminum specimen stub covered with a double-sided carbon adhesive disc (Taab, Berks, UK). Samples were sputter-coated with chromium (5–7 nm thickness) prior to imaging (Quorum Q150T ES East Grinstead, West Sussex, UK) using an acceleration voltage of 10 kV.

### 2.4. Extraction of Phenolic Compounds

A total of 500 microliters of M2 (corresponding to 0.593 g), M5 (corresponding to 0.646 g), and E (corresponding to 0.669 g) were added to a water/MeOH (80:20, *v*/*v*) solution in a 1:100 (*v*/*v*) ratio. The solutions were kept under stirring (with a magnetic stirring bar) for 30 min and then sonicated for an additional 30 min. Afterward, the solutions were centrifuged at 1790× *g* for 15 min at 20 °C. No precipitate was observed, and a 20-fold dilution with the same hydro-organic solution was conducted before the spectrophotometric assays. Finally, 100 µL of the diluted solution was used for the selected spectrophotometric assays.

Samples Syl-M2, Syl-Me, and Syl-E were solubilized in water at a 1.0 mg/mL concentration. The suspensions thus obtained were kept under stirring (with a magnetic stirring bar) for 30 min and then sonicated for an additional 30 min. Afterward, the suspensions were centrifuged at 1790× *g* for 15 min at 20 °C. Finally, an aliquot of supernatant (100 µL for each assay) was submitted to the selected spectrophotometric assays without any further dilution.

Samples Lip-Syl-M2, Lip-Syl-Me, and Lip-Syl-E were solubilized in water/MeOH (50:50, *v*/*v*) solution at a 1.0 mg/mL concentration. The suspensions thus obtained were kept under stirring (with a magnetic stirring bar) for 4 h, and the suspensions thus obtained were centrifuged at 1790× *g* for 15 min at 20 °C. Finally, an aliquot of supernatant (100 µL for each assay) was submitted to the selected spectrophotometric assays without any further dilution.

The same procedures were also applied on samples stored at 22 and 37 °C and 75% relative humidity (RH) for 1 month.

In the following methods used to determine the total phenol content and the radical scavenging capacity, the term “extract” is referred to either the diluted solutions from the M2, M5, and E samples or to the extracts from Syl-M2, Syl-M5, Syl-E, Lip-Syl-M2, Lip-Syl-M5, and Lip-Syl-E.

### 2.5. Total Phenol Content (TPC)

The total phenol content (TPC) of each extract was determined in triplicate according to the Folin–Ciocalteu method described in [20,21] with only a few modifications. The Folin–Ciocalteu reagent was diluted 10-fold with water. A definite volume of extract (0.1 mL) was mixed with 0.75 mL of the diluted Folin–Ciocalteu reagent and incubated in the dark for 10 min at room temperature (RT). Then, 0.75 mL of 2% sodium carbonate (*w*/*v*) aqueous solution were added. The mixture was kept in the dark for 3 h before measuring the absorbance at 765 nm. The content of total phenolics was determined by using a standard curve prepared with gallic acid (GA) solutions previously treated in the same way as for the real samples. Therefore, results were expressed as mg of gallic acid equivalents (GAE)/mg matrix. The matrix was different according to the sample under investigation: phytocomplex or formulation.

All the analyses were performed at 25 °C with a Varian Cary 100 (Varian Inc., Palo Alto, CA, USA) dual beam, dual chopper spectrophotometer.

### 2.6. Ferric Reducing Antioxidant Power (FRAP) Assay

The reducing power was determined in triplicate according to the method described in [20,21] with only a few modifications. The FRAP reagent was prepared as follows: 10.0 mL of a TPTZ solution (10 mM) in HCl (40 mM) were mixed with 10.0 mL of a FeCl_3_ aqueous solution (20 mM), and 100 mL of NaOAc (300 mM, pH 3.6). For the determination of the antioxidant activity, 1.5 mL of FRAP reagent was mixed with 100 µL of bidistilled H_2_O and 100 µL of the sample extract. The reaction mixture was allowed to stand for 4 min at RT before measuring the absorbance at 593 nm. The total antioxidant capacity (TAC) values were determined from a calibration curve prepared with Trolox standard solutions, previously treated by applying the same procedure as for the real sample. The antioxidant capacity of the sample was expressed as mg of Trolox equivalents/mg matrix. The matrix was different according to the sample under investigation: phytocomplex or formulation.

All the analyses were performed at 25 °C with a Varian Cary 100 (Varian Inc., Palo Alto, CA, USA) dual beam, dual chopper spectrophotometer.

### 2.7. Radical Scavenging Capacity by DPPH Method

The radical scavenging capacity was measured in triplicate by using the DPPH method as described in [21] with only a few modifications.

DPPH was progressively solubilized in HPLC-grade EtOH until a concentration producing an absorbance of 0.65 (±0.02) at 517 nm was reached. As a result, a 0.025 mg/mL solution was identified having these characteristics. Approximately 2.5 h were necessary to stabilize the above absorbance value at a temperature of 4 °C. Afterward, a volume of 0.05 mL of extract was added to 2.95 mL of DPPH solution. The absorbance was determined at 517 nm after 30 min of incubation in the dark at RT.

The antioxidant capacity of the sample was expressed as mg of Trolox equivalents/mg matrix and determined from a calibration curve prepared with Trolox solutions previously treated by applying the same procedure as for the real sample. The matrix was different according to the sample under investigation: phytocomplex or formulation.

All the analyses were performed at 25 °C with a Varian Cary 100 (Varian Inc., Palo Alto, CA, USA) dual beam, dual chopper spectrophotometer.

### 2.8. Cell Culture

Epithelial cell line HT29 (ATCC^®^ HTB-38, https://www.atcc.org/, accessed on 31 March 2021) was maintained in RPMI medium 1640 (Gibco) supplemented with glucose (25 mM), sodium pyruvate (1 mM), penicillin/streptomycin (100 µg/mL), and 10% FBS. HT29 cells were plated in 25 cm^2^ flasks and incubated at 37 °C with 5% CO_2_.

### 2.9. Evaluation of Cell Survival

Cell viability was evaluated by Trypan Blue exclusion test [22]. HT29 cells were plated in 12-wells plates and treated with the formulated compounds Syl-E or Lip-Syl-E at different concentrations, ranging from 0.625 µg/mL to 5 µg/mL, for 24 h. A group of cells were treated with vehicle alone (MeOH 10 µg/mL) for 24 h, as control. Adherent cells were detached with 800 µL of a sterile-filtered solution of trypsin-EDTA (0.25% concentrated), spun at 300× *g* for 10 min, and resuspended in 2 mL of RPMI medium. An aliquot of this suspension was mixed with Tripan Blue Solution 0.4% (1:10 dilution), and after 5 min of incubation at RT, a drop of the Trypan Blue/cell mixture was applied to a hemacytometer. Unstained (viable) cells were counted with the optical microscope (Eurotek by Orma, Milan, Italy).

To further confirm levels of cell survival in the presence of formulated compounds Syl-E or Lip-Syl-E, apoptosis was assessed by flow cytometry as described elsewhere [23]. Briefly, after cultures, cells were centrifuged for 10 min at 300× *g*, and the pellets were gently resuspended in 0.5 mL of hypotonic PI solution (50 μg/mL propidium iodide in 0.1% sodium citrate plus 0.1% Triton X-100; Sigma) for 2 h in the dark at 4 °C. PI fluorescence of individual nuclei was measured by Coulter Epics XL-MCL Flow Cytometer (Beckman Coulter, Brea, CA, USA). Data were analyzed using FlowJo 10.4 software (TreeStar, Ashland, OR, USA).

### 2.10. Evaluation of Antioxidative Activity

The antioxidative activity of formulated compounds was assessed by 2′,7′-dichlorodihydrofluorescein diacetate (DCFDA) test. DCFDA is deacetylated and oxidized to 2′,7′-dichlorodihydrofluorescein (DCF), which is fluorescent. The fluorescence generated is proportional to the amount of DCFDA oxidated to DCF. After 24 h of treatment with formulated compounds, cells were incubated with 5 μM DCFDA (ThermoFisher, dissolved in DMSO) in a FACS tube for 10 min at 37 °C. The fluorescence was detected using the Attune ATTUNE NxT flow cytometer (ThermoFisher, Waltham, MA, USA). Data were analyzed using FlowJo 10.4 software (TreeStar, Ashland, OR, USA) and normalized compared to controls (vehicle alone).

### 2.11. Statistical Analysis

The data obtained from the HPLC analysis of the phenolic extracts (2 analytical repetitions) and the data obtained from the Folin–Ciocalteu assay applied to determine the TPC (2 extraction processes, 3 analytical repetitions) were analyzed using a one-way ANOVA and a Tukey’s post hoc test. Results were considered significant for *p* < 0.05.

All statistical analysis for biological evaluations were performed with Prism 7.0 (GraphPad, San Diego, CA, USA). Results shown in figures are representative of at least 3 independent experiments. The two-tailed unpaired Student’s *t*-tests were used for statistical comparison (* *p* < 0.05; ** *p* < 0.005; *** *p* < 0.0005; **** *p* < 0.0001).

## 3. Results and Discussion

### 3.1. HPLC Characterization of Phenolic Extracts

The three phenolic extracts (M2, M5, and E) were subjected to qualitative-quantitative characterization as reported above by HPLC/DAD analysis (Table 1). The most abundant phenolic species in the extracts was the dialdehydic forms of elenolic acid linked to 3,4-DHPEA (3,4-DHPEA-EDA), ranging between 66.5 and 81% of the total phenolic fractions, followed by hydroxytyrosol (3,4-DHPEA), accounting for 12–22% in the three extracts. Not negligible amounts of verbascoside and tyrosol (p-HPEA) were generally detected, while other compounds such as p-HPEA-EDA and vanillic acid were present only in extract E. The composition of that “phytocomplex”, whose bioactivity was largely described and recognized, confirms the presence in OMWWs of phenols naturally contained also in virgin olive oil, while verbascoside, due to its polarity, was only present in the raw olive fruits and by-products [13,24].

### 3.2. Phenolic Extract Inclusion Complexes

Syloid^®^ AL-1 was chosen for its low cost and its previous use in food and pharmaceutical products [25,26,27,28]. Syloid^®^ AL-1 is a multifunctional excipient characterized by high porosity and, therefore, a high surface area (~680 m^2^/g). In this work, Syloid^®^ AL-1 was used as a carrier for the phenolic extracts thanks to its high adsorptive feature. As stated in the introduction, phenolic extracts are of great interest for their application in different fields such as food and feed but are characterized by intrinsic instability. Mesoporous silica is able to prevent degradation adsorbing the damaging moisture and improve stability during storage. Another interesting aspect of Syloid^®^ AL-1 is its ability to effectively convert liquids in free-flowing powders that are easier to manage in the successive steps of the final product preparation. Phenolic extracts would benefit from this last property since they are very viscous liquids that can difficultly be dosed and handled. This intrinsic feature of phenolic extracts was an issue when spray-drying was used to encapsulate this viscous liquid in whey protein or maltodextrin [29]. In fact, in some conditions, the powder obtained was slurry or sticky and could not be used. Besides, spray-drying required a case-by-case optimization that was time-consuming [30], while the inclusion process in mesoporous material performed in this study was a straightforward procedure.

The solvent evaporation method was chosen to load the phenolic extracts in Syloid^®^ AL-1 because this procedure allows controlling the quantity of extract loaded in the mesoporous silica [17]. Though this process requires the use of organic solvent, according to the European Pharmacopoeia, ethanol is a class 3 solvent [31] that corresponds to a solvent with low toxic potential. Moreover, ethanol can be easily and rapidly eliminated using rotary evaporation. The amount of phenolic extract to be loaded (phenolic extract:Syloid^®^ AL-1, 30:70, *w*/*w*) was chosen on the basis of the values reported in the literature for different drugs and Syloid^®^ AL-1, and other mesoporous silicas having comparable surface area [26,32,33]. A theoretical loading of 30% (*w*:*w*) allows obtaining a final product where the phenolic extract is completely adsorbed and included in the pores of the mesoporous silica. As such, phenolic extract inclusion complexes can be exploited for their antioxidant properties in food products to replace preservatives or to obtain a fortified food product. For this last application, the inclusion complex should be added to the food product just before it is eaten. In fact, once in contact with liquids and, for instance, water, phenolic extracts will be rapidly released from the silica.

A lipid mixture was used to coat the inclusion complexes to hinder early phenolic extract release when the inclusion complex is in contact with water. In particular, the lipid mixture of lauric and stearic acid in a 77:33 wt ratio was chosen to obtain its fusion at body temperature and consent phenolic extract solubilization in gastric fluid. This strategy would allow adding the lipid-coated inclusion complex in food, such as yogurt, hindering its release until the product is eaten.

**Figure 1:**
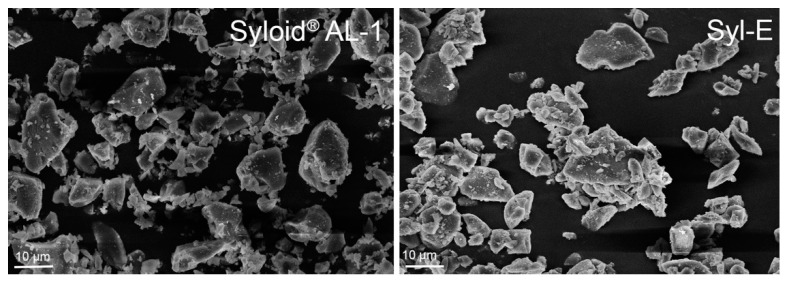
SEM photomicrographs of Syloid^®^ AL-1 and Syl-E inclusion complex.

SEM photomicrographs show that Syl-E particles have a morphology similar to that of Syloid^®^ AL-1 (Figure 1). As previously reported [18], Syloid^®^ AL-1 powder is composed of irregular particles with an average particle size corresponding to the product specifications. Syloid^®^ AL-1 mean particle size is lower than 10 µm and this consents to hypothesize the use of the inclusion complex as is. In fact, their perception of ingestion should be marginal, or they may not be perceived at all. To confirm this, we can cite the example of chocolate that will be perceived as sandy if cocoa and sugar particles are larger than ~25–35 μm [34].

### 3.3. Total Phenol Content

A preliminary evaluation of the total phenol content (TPC) of the three extracts was determined by the use of the original Folin–Ciocalteu method with only a few modifications [20,21] (Table 2, entries a–c).

The Folin–Ciocalteu assay is well-recognized for its appreciable cost-effectiveness, rapidity, and simple execution, even though one of its major intrinsic limits is the impossibility to derive neither qualitative nor quantitative information of the single phenols belonging to the phytocomplex under investigation. Indeed, these species are cumulatively quantified by using gallic acid as a surrogate standard. Nevertheless, this method can be conveniently used for comparative purposes, mostly in the cases of phenol mixtures containing the same chemical sub-classes. Based on all the above, the Folin–Ciocalteu test was performed in the present study for TPC evaluations.

TPC values listed in Table 2 indicate the lowest value for sample M2 and the highest for sample E (Table 2, entries a–c). Each data are significantly different from each other (*p* < 0.05), and phenolic extract can be classified according to the TPC as follows: E > M5 > M2. These differences are consistent with the results of the HPLC-DAD analysis and show how the phenolic concentration in the OMWWs and their corresponding extracts can vary largely due to the processing of the olive batches and slightly to the execution of the recovery.

In order to confirm the accuracy of these results, the extraction ability of the original water-methanol-based solvent was compared with that of an alternative solution made up of net ethanol. Only negligible differences were observed (data not shown) between the two extraction solvents, thereby indicating that the selected binary hydro-alcoholic system was suitable for the purpose of this study.

Setting aside the extract with the highest phenol concentration (namely, extract E) for the following biological evaluations, only the inclusion compounds obtained from extracts M2 and M5 were submitted to further investigations aimed at measuring the phenol content after the applied technological processes and following the different storage conditions.

The phenol content determined in Syl-M2 and Syl-M5 (Table 2, entries d and e) was in strict accordance with the amount of extract loaded, ultimately indicating that the preparation procedure did not alter the TPC. By going into the detail of the results, about 31% of the overall phenol content (see Section 2.4. for details on the calculation of this value) was estimated in Syl-M2 (Table 2, entry d) and 34% in Syl-M5 (Table 2, entry e).

For Lip-Syl-M2 and Lip-Syl-M5, the phenol content was about 18% and 16%, respectively (Table 2, entries g and h, respectively), in line with the phenolic extract theoretical loading for Lip-Syl-M5 while it was slightly higher for Lip-Syl-M2. The same trend was also observed for Lip-Syl-E (Table 2, entry i), which showed a higher phenol content than the extract percentage loaded. This behavior can be explained by the reactivity of the Folin–Ciocalteu reagent towards lauric and stearic acid. In fact, the phosphomolybdic/phosphotungstic reagent can potentially react with any reducing species in alkaline conditions and not only with phenolic compounds [35]. Fatty acids can be easily oxidized and are, therefore, reducing species that react with the Folin–Ciocalteu reagent leading to a small overestimation of the TPC [36,37].

Two consecutive extractions were performed on samples Syl-M2 and Syl-M5, and the application of the Folin–Ciocalteu assay revealed that the first extraction was almost complete, with about only 1% of phenols extracted with the second step.

From a look at the data listed in Table 2 (entries a–i), it is readily evident the great accordance (RSD% ≤ 0.13) among the results obtained from three separate analyses on matrixes M2, M5, and E as well as on their inclusion Syl and Lip-Syl complexes. Instead, a wide variability (0.64 ≤ RSD% ≤ 14.41; Table 2, entries j–q) was found for the samples submitted to different storage conditions, with the spreadiest results calculated for samples Lip-Syl-M2 and Lip-Syl-M5 at 37 °C and 75% RH.

As far as the stability tests are concerned, the TPC remained nearly unchanged in Syl-M2 and Syl-M5 irrespective of the temperature, and the RH fixed over the storage period (Table 2). Indeed, after the storage at 22 °C and 75% RH, a TPC of about 35% was estimated for both Syl-M2 and Syl-M5 (entries j and k, respectively), while a slight decrease in terms of phenol content was measured after the storage at 37 °C and 75% RH: 26% of the TPC for Syl-M2 and 29% for Syl-M5 (entries l and m, respectively). The one-way ANOVA and Tukey’s post hoc test suggest that there was no significant difference between the TPC values of Syl-M2 before and after storage at 22 or 37 °C and 75% RH. The same conclusion was obtained for Syl-M5 values before and after 1-month storage at 22 °C and 75% RH, while a small but significant decrease of the TPC was recorded after storage at 37 °C and 75% RH. The last data should, however, be balanced by the very small difference between the absolute different and critical range values of the post Tukey test.

This high stability, both at 22 and 37 °C and 75% RH, confirmed the ability of Syloid^®^ AL-1 to absorb moisture protecting the phenolic extract from degradation in harsh humidity conditions.

A higher variability was measured for the two products obtained after the lipid coating: Lip-Syl-M2 and Lip-Syl-M5. A TPC of about 22% and 24% after the storage at 22 °C and 75% RH (Table 2, entries n and o) was estimated for Lip-Syl-M2 and Lip-Syl-M5, respectively. Unexpectedly, significantly higher TPC values (*p* < 0.05) were obtained after storage at 22 °C and 75% RH with respect to the starting lipid-coated inclusion complexes. In the more drastic storage conditions (37 °C, 75% RH, 1 month), an abrupt decline turned out for these two samples: 5% of TPC for sample Lip-Syl-M2 and 7% for Lip-Syl-M (entries p and q). As reported above, lipids are sensible to oxidation, autoxidation being the main process responsible for their degradation [38,39]. Lipid autoxidation commonly accelerates with the increase of temperature and is responsible for the significant reduction of the TPC values (*p* < 0.05). However, these data are not incompatible with the aim of the research to stabilize the phenolic extract until its use. The lipid-coated inclusion complexes will not be exposed to these drastic conditions but to milder ones, in particular in terms of temperature (e.g., 4 °C for yogurt storage). Therefore, the lipid coating is effective in preventing premature phenolic content release in contact with water (e.g., aqueous food matrixes) until it will be ingested.

It is important to emphasize that, irrespective of the starting extract (that is, either M2 or M5), appreciable repeatability of the formulation process was observed. The Folin–Ciocalteu assay was also applied to study the inclusion compounds Syl-E and Lip-Syl-E, which were prepared for the following biological evaluations. A TPC value of about 24% and 15% (Table 2, entries f and i, respectively) was measured, further confirming the satisfactory quality of the inclusion preparation.

In order to appraise whether the technological processes can have a negative impact on the total antioxidant capacity (TAC) of the formulated compounds, two spectrophotometric tests were applied on samples Syl-E and Lip-Syl-E. In particular, the FRAP and the DPPH assays were selected, with the former being exclusively based on an electron-transfer (ET) mechanism, while the latter is able to determine the antioxidant capacity of compounds activating either with an ET or a hydrogen atom transfer (HAT) mechanism, or both [20,21]. TAC values evaluated with the above-mentioned assays are listed in Table 3 and Table 4.

Very importantly, data reported in Table 3 and Table 4 reveal that the formulation processes do not affect the TAC of the phenolic pool.

### 3.4. Analyses of Biological Properties of Syl-E and Lip-Syl-E Formulated Compounds

**Figure 2:**
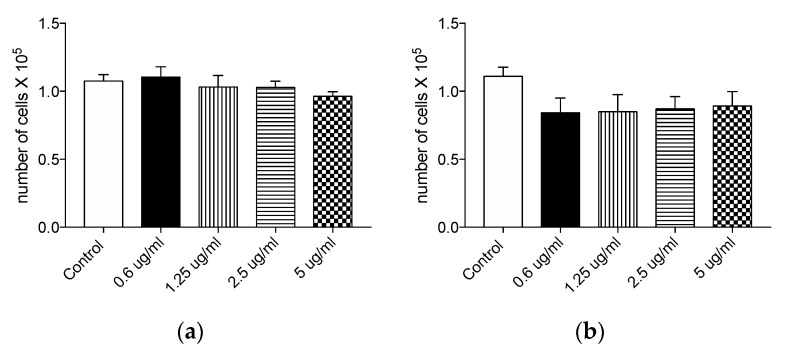
Syl-E and Lip-Syl-E phenolic formulated compounds do not affect cell viability upon 24 h of treatment. Graphs represent the mean number of viable cells upon 24 h treatment with Syl-E (**a**) or Lip-Syl-E (**b**) formulated compounds at different concentrations (as indicated on the x-axis), assessed by the Trypan Blue test. Data are derived from three independent experiments. No statistical differences between experimental groups were found.

The potential toxicity of Syl-E and Lip-Syl-E formulated compounds was tested in human cells by assessing compound-dose response measures of cellular viability. A HT29 cell line and an intestinal-derived epithelial cell line, was treated for 24 h with different doses of Syl-E or Lip-Syl-E formulations (concentrations ranging from 0.625 µg/mL to 5 µg/mL). At the end of exposure, cells were counted by Trypan Blue exclusion test using an optical microscope. Results shown in Figure 2 indicate that none of the concentrations used for the two formulations, Syl-E or Lip-Syl-E, was toxic for HT29 cells after administration for 24 h (Figure 2a,b respectively).

**Figure 3:**
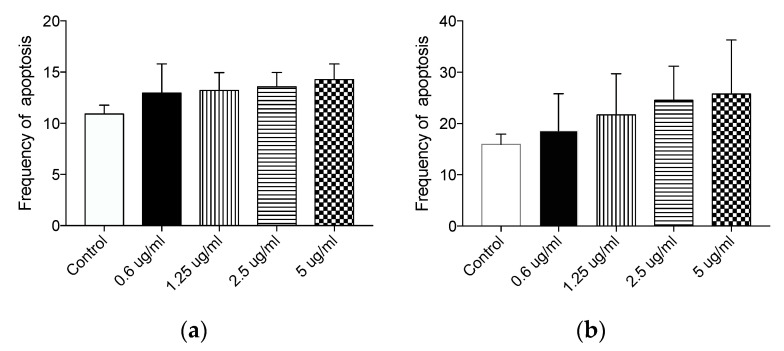
Syl-E and Lip-Syl-E phenolic formulated compounds do not affect apoptosis upon 24 h of treatment. Graphs represent the mean of apoptotic cells upon 24 h treatment with Syl-E (**a**) or Lip-Syl-E (**b**) formulated compounds, at different concentrations (as indicated on the x-axis), assessed by Propidium Iodide staining followed by flow cytometry analysis. Data are derived from three independent experiments. No statistical differences between experimental groups were found.

The absence of toxicity was further confirmed by measuring the apoptotic rate of HT29 cells exposed to different doses of Syl-E or Lip-Syl-E by flow cytometry analysis upon PI staining: no significant differences were detected in the percentage of apoptosis in treated cells compared to controls (Figure 3a,b).

In order to evaluate the antioxidant effect of formulated compounds, the amount of ROS (Reactive Oxygen Species) produced by HT29 cells was measured upon 24 h exposure to the two formulations at non-toxic concentrations by flow cytometry analysis.

**Figure 4:**
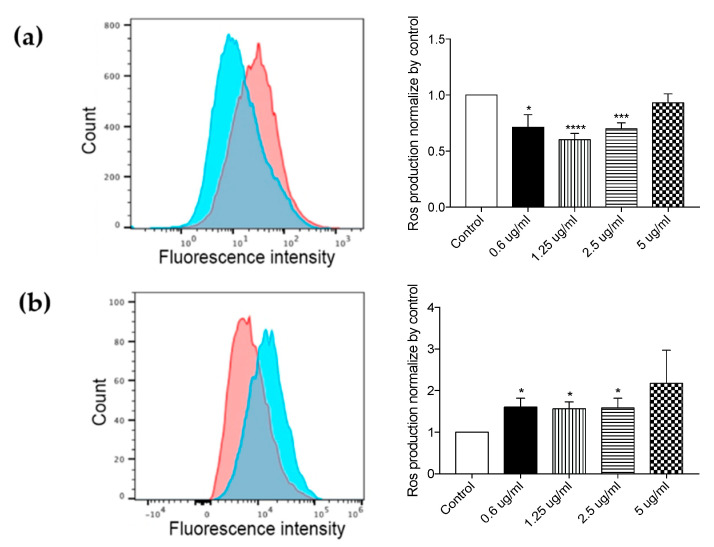
The Syl-E, but not Lip-Syl-E, compound shows antioxidant activity in the HT29 cell line 24 h after treatment. Histograms on the left of the panel are representative of flow cytometric analysis for the evaluation of ROS production on the HT29 cell line evaluated by DCFDA administration, with (blue histogram) or without (red histogram) treatment with Syl-E (**a**) or Lip-Syl-E (**b**) formulated compounds. The graph on the right represents the means of the measure of ROS production of different concentrations tested (as indicated on the x-axis). Data are derived from three independent experiments. Statistical analysis was performed using the unpaired Student’s *t*-test (* *p* <0.05; *** *p* <0.0005; **** *p* < 0.0001).

Data represented in Figure 4a indicate that groups of cells treated with 1.25 µg/mL and 2.5 µg/mL of Syl-E formulation have a significant antioxidant effect compared with controls. On the contrary, HT29 cells treated with Lip-Syl-E formulation showed an increased production of ROS compared with untreated cells (Figure 4b). On this basis, we can assume that the lipid component of Lip-Syl-E exerts a pro-inflammatory action, ultimately affecting the antioxidant properties of the phenolic complex.

## 4. Conclusions

The two different inclusion complexes produced in the study (lipid- and non-lipid-coated) showed distinctive, interesting features. The lipid-coated complex stability profile was less promising under drastic conditions. However, it could be employed as a suitable stabilizing strategy for the preparation of phenolic-based fortified liquid or semi-liquid food matrices stored at 4 °C. The non-lipid-coated complex was demonstrated to be very stable and maintained all the phenolic extract properties (TPC, TAC, in vitro antioxidant activity). Therefore, its use can be thought to replace or reduce the number of conventional preservatives added to the food product. These assumptions are further corroborated by in vitro cytocompatibility tests using an intestinal-derived epithelial cell line demonstrating that these formulations are not toxic and maintain antioxidant properties of phenolic extracts.

## Figures and Tables

**Table 1:** Phenolic composition (mg/g) of the extracts obtained from OMWWs.

Compounds	E		M5		M2	
	Average	RDS%	Average	RDS%	Average	RDS%
3,4-DHPEA	70.40 ^a^	0.10	109.00 ^b^	0.50	105.70 ^c^	0.30
p-HPEA	11.40 ^a^	0.10	20.20 ^b^	0.01	23.90 ^c^	0.03
Vanillic acid	0.78	0.01	n.d.		n.d.	
Verbascoside	25.60 ^a^	1.90	33.20 ^b^	1.50	30.90 ^a,b^	1.30
3,4-DHPEA-EDA	479.30 ^a^	0.90	325.40 ^b^	1.30	318.50 ^c^	0.50
p-HPEA-EDA	4.90	0.80	n.d.		n.d.	
Sum of phenolic fractions	592.40 ^a^	0.70	487.90 ^b^	0.90	479.00 ^b^	0.40

The statistical significance of differences is marked with letters. Within a raw, means without a common superscript differ (*p* < 0.05).

**Table 2:** Phenol content and percentage of inclusion.

Sample	Entry	Phenol Content (mg eq. GA/mg Matrix ^y^)		Content %
		Average	RSD%	
M2	a	0.24 ^b^	0.04	100
M5	b	0.22 ^c^	0.06	100
E	c	0.28 ^a^	0.02	100
Syl-M2	d	0.08 ^d,e^	0.09	31
Syl-M5	e	0.08 ^f^	0.04	34
Syl-E	f	0.07	0.09	24
Lip-Syl-M2	g	0.04 ^h^	0.11	18
Lip-Syl-M5	h	0.04 ^k^	0.13	16
Lip-Syl-E	i	0.04	0.11	15
Syl-M2, 22 °C, 75% RH	j	0.09 ^d^	10.84	35
Syl-M5, 22 °C, 75% RH	k	0.08 ^f^	3.97	35
Syl-M2, 37 °C, 75% RH	l	0.06 ^e^	4.34	26
Syl-M5, 37 °C, 75% RH	m	0.07 ^g^	8.35	29
Lip-Syl-M2, 22 °C, 75% RH	n	0.05 ^i^	1.74	22
Lip-Syl-M5, 22 °C, 75% RH	o	0.05 ^l^	0.64	24
Lip-Syl-M2, 37 °C, 75% RH	p	0.01 ^j^	11.63	5
Lip-Syl-M5, 37 °C, 75% RH	q	0.02 ^m^	14.41	7

^y^ The matrix was different according to the sample under investigation: phytocomplex or formulation. The statistical significance of differences is marked with letters. Within an inclusion complex stored in different conditions, means without a common superscript differ (*p* < 0.05). Analysis has been performed separately on the different inclusion complexes stored in different conditions since the phenolic extracts are significantly different from one another. ^a,b,c^ are for M2, M5 and E. ^d,e^ are for Syl-M2 in different conditions. ^f,g^ are for Syl-M5 in different conditions. ^h,i,j^ are for Lip-Syl-M2 in different conditions. ^k,l,m^ are for Lip-Syl-M5 in different conditions. Analysis was not performed on Syl-E and Lip-Syl-E since they were not stored in different conditions.

**Table 3:** Total antioxidant capacity measured with the FRAP assay and percentage of retained antioxidant capacity in the E-based formulations.

Sample	Entry	FRAP Assay (mg eq. Trolox/mg Matrix ^y^)		Content %
		Average	RDS%	
E	a	0.35	0.05	100
Syl-E	b	0.09	0.23	25
Lip-Syl-E	c	0.05	0.17	14

^y^ The matrix was different according to the sample under investigation: phytocomplex or formulation.

**Table 4:** Total antioxidant capacity measured with the DPPH assay and percentage of retained antioxidant capacity in the E-based formulations.

Sample	Entry	DPPH Assay (mg eq. Trolox/mg Matrix ^y^)		Content %
		Average	RSD%	
E	a	0.21	0.49	100
Syl-E	b	0.05	0.39	24
Lip-Syl-E	c	0.03	0.62	15

^y^ The matrix was different according to the sample under investigation: phytocomplex or formulation.

## Data Availability

Data is contained within the article.
